# Growth, carcass traits, immunity and oxidative status of broilers exposed to continuous or intermittent lighting programs

**DOI:** 10.5713/ajas.20.0328

**Published:** 2020-08-21

**Authors:** Mahmoud M. Abo Ghanima, Mohamed E. Abd El-Hack, Mohammed Sh. Abougabal, Ayman E. Taha, Vincenzo Tufarelli, Vito Laudadio, Mohammed A. E. Naiel

**Affiliations:** 1 Department of Animal Husbandry and Animal Wealth Development, Faculty of Veterinary Medicine, Damanhour University, Damanhour 22511, Egypt; 2 Department of Poultry, Faculty of Agriculture, Zagazig University, Zagazig 44511, Egypt; 3 Department of Animal Production, Faculty of Agriculture, Al-Azhar University, Cairo 11754, Egypt; 4 Department of Animal Husbandry and Animal Wealth Development, Faculty of Veterinary Medicine, Alexandria University, Rasheed, Edfina 22758, Egypt; 5 Department of DETO, Section of Veterinary Science and Animal Production, University of Bari ‘Aldo Moro’, 70010 Bari, Italy; 6 Department of Animal Production, Faculty of Agriculture, Zagazig University, Zagazig 44511, Egypt

**Keywords:** Lighting Program, Oxidative Status, Innate Immunity, Broilers, Performance

## Abstract

**Objective:**

An experiment was conducted to investigate the continuous and intermittent lighting program effects on terms of the productive performance, carcass traits, blood biochemical parameters, innate immune and oxidative status in broiler chicks.

**Methods:**

A total of 600 Cobb-500 one day old chicks were randomly allocated into six equal groups (100 chicks per treated group with five replicates of 20 chicks each) based on lighting program; 22 continuous lighting (22 C), 11 h lighting+1 darkness twice daily (11 L/1 D), 20 h continuous lighting (20 C), 5 h lighting+1 darkness four times daily (5 L/1 D), 18 h continuous lighting (18 C) and the final group subjected for 3 h lighting+1 h darkness six times daily (3 L/1 D). The experimental period lasted 42 days.

**Results:**

Compared with those under the intermittent light program, broiler chicks exposed to continuous lighting for 22 h had significant improvement in live body weight and carcass (dressing and breast percentage) measured traits. Though reducing lighting hours significantly reduced feed intake and feed conversion ratio values. Different lighting programs revealed no significant effect on all blood biochemical parameters. Oxidative stress and innate immunity parameters significantly enhance by reducing lighting hours (3L/1D).

**Conclusion:**

The findings suggest that reducing lighting hours up to 3L/1D would be more useful in enhancing feed efficiency, innate immunity, and oxidative status compared with continuous lighting programs on broilers.

## INTRODUCTION

Globally, demand for producing high-quality broilers with lower costs has increased the emphasis on enhancing bird health and immunity to optimum performance. Rapidly growing broiler chickens, especially in early stages, are associated with several health problems such as sudden death syndrome (SDS), ascites and skeletal abnormalities [1]. Duration of lighting plays a significant role in the health of broilers [2]. Several studies have investigated the negative effects of low photoperiod regime and light intensity on broiler’s carcass traits, meat quality, skeletal disorders, as well as innate immunity and health [3,4]. Several studies were performed [5,6] confirming some health problems associated with fast growth reduced with extended daily dark periods (6L/18D) in early aged broilers (from 3 till 14 days old). Likewise, Scott [1] revealed enhanced growth occurred with increasing day length (23 h) for 14 up to 42 days broiler chickens. Therefore, using continuous lighting (CL) programs for 23 and 22 h results in maximal growth performance as chicks will consume more feed but indeed it will also result in an increased incidence of metabolic diseases and other high-performance problems [7,8].

Broiler producers extensively use an intermittent lighting (IL) programs on broiler farms [9]. According to Buyse et al [8] findings, the IL programs have a promising effect on feed conversion. These findings may be due to reducing bird maintenance, fat deposition and activity. Besides, Apeldoorn et al [9] revealed that reduced feed intake while maintaining growth rate were correlated with enhancement feed conversion under IL programs. The higher metabolizable and growth energy utilization ratio, as well as a lower activity heat production, was correlated with lower feed efficiency. Many researchers studied the impact of using IL for the same hours of continuous or less hours on performance and revealed improved in the final body weight (BW) linked with reduced feed conversion ratio (FCR) values and enhancing health status. The reduction of activity during the hours of darkness may result in lower heat production, higher feed efficiency or both [10]. Also, lowering ascites and leg problems are correlated with applied IL programs in broiler farms [11].

Worldwide, the Cobb 500 broiler is a recent commercially available broiler strain. This strain has made an impression on the commercial poultry market and has a unique growth rate and yielding ability characteristics compared to other available strains [12]. Therefore, few studies had been performed to evaluate the effect of different lighting programs to obtain the best growth performance by reducing the drawbacks of high growth rate, feed consumption (FC) and considering maintaining the immune system in maximum growth on broilers [13].

Therefore, the propose of the current study was not only to compare the continuous and IL programs but also to evaluate their effect on growth performance, carcass traits, blood parameters, oxidative stress and innate immunity in cobb broiler.

## MATERIALS AND METHODS

All experimental procedures were followed and approved by the Ethics Committee of the Local Experimental Animal Care Committee, Department of Animal Husbandry and Animal Wealth Development, Faculty of Veterinary Medicine, Damanhour University.

### Experimental design, managements and feeding regime

The present study was carried out at the Research Poultry Farm, Faculty of Veterinary Medicine, Damanhour University, Egypt. A total number of 600 one-day-old of both sexes Cobb-500 broiler chicks were purchased from El-Watania Hatcheries, km 59, Alexandria - Cairo Desert Road, Alexandria, Egypt. Chicks were individually weighed to make uniform replicate groups. The chicks were distributed in a completely randomized design into six equal experimental groups (n = 100) with five replicates (20×5). The six groups were as follows: T1, birds subjected to 22 continuous lightings (CL22); T2, birds subjected to 11 h lighting and 1 h darkness (11 L/1 D) twice daily (IL22); T3, birds subjected to 20 h continuous lighting (CL20); T4, birds subjected to 5 h lighting and one h. darkness (5 L/1 D) 4 times daily (IL20); T5, birds subjected to 18 h continuous lighting (CL18); and T6, birds subjected to 3 h lighting and 1 h darkness (3 L/1 D) six times daily (IL18).

The facility was equipped with supplementary pan feeders and drinkers for the brooding period and then birds were moved to the cages. The birds had free access to feed and water for *ad libitum* consumption. For the first three weeks, chicks were fed on starter ration (2,900 kcal ME/kg, 23% crude protein [CP] from 0 to 21 days) and followed by grower ration (3,200 kcal ME/kg, 21.5% CP from 21 to 42 days) for the remaining period of the experiment as presented in Table 1. Diets were manufactured by the El-Fagr company for the feed industry (Al Nubarya, El Bohira, Egypt).

### Management and vaccination

Chicks were brooded in floor pens (covered with wood shaving litter) during the first two weeks with using supplemental feeders and drinkers and then moved to cages, each replicate housed in 2 separate pens (each pen 100 cm length×90 cm width×45 cm height). The cages provided with separate lines of nipple drinkers for each pen and separate feeders. Chicks were raised under identical hygienic, managerial, and environmental conditions throughout the study conforming to manual guide recommendation for the strain. Briefly, the temperature was set constant 33°C at the birds’ level during first three days of age then gradually reduced with 1°C per two days until 25°C at 21 days and then remained at 25°C up to the end of the rearing period. The lighting schedule was applied, as mentioned before, from hatch to the end of the rearing period (42 days). The intensity of measured light in the middle of the room was ranged between 5 and 10 lux. Routine vaccination schedule was administered and necessary medication when needed based on diagnoses and symptoms shown by the birds.

### Growth performance

During the experimental period, all birds were subjected to the same method of data collection. Chicks in each replicate were individually weighed at weekly intervals with electric balance until 6 weeks of age. Weighing of the birds was done every week in the early morning before receiving any feed or water. Also, weekly FC, total feed consumption (TFC), and dead birds (if any) were recorded.

Growth traits include the determination of BW, body weight gain (BWG), and FCR, mortality (%) and livability (%). After calculation of livability % and FCR performance index of European Production Efficiency Factors (EPEF) were used to evaluate the growing performance index of broilers as suggested by Aviagen [14]. The EPEF were calculated according to the following formula: (Livability [%]×BW [kg]/Age [d] ×FCR)×100.

$$\text{EPEF}=\frac{\text{Livability}\%\times \text{BW }(\text{kg})}{\text{Age }(\text{d})\times \text{FCR}}\times 100$$

### Carcass traits

At the end of the trial (42 days), 20 birds (10 males and 10 females) from each group were randomly chosen around the average weight of the group to determine the carcass characteristics and internal organs. Birds were slaughtered with a knife (Halal Method), allowed to bleed for 150 s, scalded at 60°C×90 s, de-feathered and manually eviscerated. Following evisceration, all carcasses were chilled in cold water for 15 min. Hot carcass, breast, thigh, shoulder, left filet, liver, heart, gizzard, intestine, and abdominal fat were weighed. The blood, viscera, lungs, limbs, head, and neck were termed as the offal’s and they were discarded. The abdominal fats in the pelvic and abdominal cavity were collected separately from the carcass and weighted. The technological division of the carcass was performed and calculated according to Xie et al [15]. Breast, thigh, shoulder, left filet, liver, heart, gizzard, intestine, and abdominal fat were expressed as a percentage of the carcass weight. Dressing percentage, after weighing warm carcass, was calculated according to Raza et al [16].

### Blood biochemical parameters

The blood samples (5 samples from each replicate) were collected from the wing vein at 42nd day of the experiment. Blood tubes were placed at a slant position at room temperature for 30 min and then separated through centrifugation at 3,000 rpm×15 min. The separated serum samples were collected, frozen and stored at −20°C until subsequent analysis of blood biochemical parameters.

Total protein and Albumin were determined using the Bio-diagnostic colorimetric kit according to the method of Apanius et al [17]. The values of globulin were calculated by subtracting the albumin values from total protein values. Serum total lipids were determined by the Total lipids kit of Bio-diagnostic according to the method of Zollner and Kirsch [18]. Serum Triacylglycerol was determined by the Triacylglycerol kit of Bio-diagnostic according to the method of Fossati and Prencipe [19]. Cholesterol was determined using the Allain et al [20] enzymatic method. Determination of alanine aminotransferase (ALT) and aspartate aminotransferase (AST) were determined colorimetry by ALT and AST kit of Bio-diagnostic according to the method of Reitman and Frankel [21]. Creatinine was colorimetric dynamic determined as described by Bartles et al [22]. While the determination of urea level was according to the Fawcett and Scott [23] method.

### Assessment of antioxidative response

The malondialdehyde (MDA) concentration concentrations in homogenates were evaluated by the method of Jo and Ahn [24]. Serum blood activity of glutathione peroxidase (GPx) was assessed by using ELISA Kit: Catalog No. DPOD-100; Quanti Chrom TM, BioAssay Systems, Hayward, CA, USA), according to Kokkinakis and Brooks [25]. Super oxide dismutase (SOD) activity was measured by the xanthine oxidase method (ELISA Kit: Catalog No. 706002; Cayman Chemical Company, Ann Arbor, MI, USA), which monitors the inhibition of nitro blue tetrazolium reduction by the sample Sun et al [26].

### Innate immunity measurements

Blood samples were collected at 42 days of age (5 samples per replicate) as mentioned above. Phagocytic activity and phagocytic index (PI) were determined as described by Kawahara et al [27]. Briefly, 15 μg *Candida albicans* culture was added to 1 mL of citrated blood from each group and incubated in the water bath at 25°C for 5 h, and then blood smears from each tube were stained with Giemsa stain. Phagocytosis was estimated by determining the proportion of macrophages which contained intracellular yeast cells in a random count of 300 macrophages and expressed as percentage of phagocytic activity (PA). The number of phagocytized organisms was counted in the phagocytic cells and called PI.

### Statistical analysis

Data obtained were analyzed by a one-way analysis of variance using Statistical Analysis System (SAS,v9) [28]. The main effect of the lighting program was the experimental unit. Means were compared by Duncan’s multiple range test [29] when a significant difference was detected. The following Proc general linear model model was used for the analysis of variance: X_ijkl_ = μ+A_j_+*e_i_*; where: X_ij_ = observational data, μ = overall mean, A_j_ = effect of lighting program, *e_i_* = random error.

## RESULTS

### Growth performance

Results of the lighting program’s effects on growth performance parameters (LBW [live body weight], BWG, FC, and FCR) are presented in Table 2. Results show that significant differences were found in all parameters studied among all groups. Broilers final body weight was significantly (p<0.01) affected by different lighting programs. Cleary, broiler group, subjected to 22 h continuous light (CL22) exhibited higher final LBW followed in order by groups which reared at 11L/1D, 20 h continuous, 5L/1D, 18 h continuous or 3L/1D light programs (IL22, CL20, IL20, CL18, and IL18). Final LBW at 6th week of age was reduced by about 5.44%, 6.79%, 8.48%, 9.14%, and 11.27% for T2, T3, T4, T5, and T6, respectively in comparison with T1 group (CL22). Equally important, the obtained BW results from chickens reared under long photoperiod 22 h. were higher than the groups reared at 20 h and 18 h, whether it is continuous or intermittent (CL or IL). Not only LBW but also BWG took the same trend where 22 h continuous light broiler group (CL22) obtained higher BWG at 6th weeks of age followed in order by groups that reared on lighting programs at IL22, CL20, IL20, CL18, and IL18, respectively.

Furthermore, regarding feed utilization, different lighting programs significantly (p<0.01) affect the TFC of all groups. It was found that broilers reared in CL programs (CL22, CL20, and CL18) consumed significantly more feed than their comparable exposed to IL programs (IL22, IL20, and IL18), respectively. At the same time, broiler groups exposed to long photoperiod for 22 h (T1 and T2) whether CL or IL consumed significantly more feed than those in medium photoperiod for 20 h (T3 and T4) or short photoperiod for 18 h (T5 or T6). Equally essential results showed that lighting programs significantly (p<0.05) affect the total FCR of all broiler groups. It was found that chickens raised in most prolonged light intervals T1 and T2 (for 22 h whether CL or IL) recorded significantly higher and lowest total FCR (1.58) values than those in medium (1.65 and 1.54 for T3 and T4) or short photoperiod for 18 h (1.55 and 1.54 for T5 or T6), respectively. Besides, broilers reared in IL programs (IL22, IL20, and IL18) recorded better FCR than others exposed to CL programs (CL22, CL20, and CL18), respectively.

### Mortality rate and European Production Efficiency

Regarding the mortality rate, the results presented in Table 2 show significant differences (p<0.01) among the experimental groups as affected by different lighting programs. Cleary, broiler group, subjected to 22 h continuous light (CL22) recorded higher mortality % followed in order by groups which reared at IL22, CL20, IL20, CL18, and IL18. Equally important, broiler groups reared under a continuous light program for 22, 20, or 18 h (CL22, CL20, and CL18) registered higher values of mortality % than those reared at an intermittent light program (IL22, IL20, and IL18). The mortality rate was reduced by about 23.46%, 11.44%, 41.06%, 41.06%, and 88.27% for IL22, CL20, IL20, CL18, and IL18, respectively in compare with broiler group exposed to CL for 22 hrs. Consequently, the opposite trend was found in livability % of experimental groups as affected by different lighting programs. Finally, the most important indicator of growth performance, as a term of EPEF, broiler group exposed to continuous light for 22 h (CL22) exhibited higher EPEI (396 pts) followed by groups which reared at IL22, CL20, IL20, CL18, and IL18 (378, 375, 377, 375, and 372 pts), respectively.

### Carcass characteristics

Results of dressing %, carcass traits and internal organs as affected by lighting programs are presented in Table 3. Results showed that, lighting program significantly (p<0.05) affected dressing %, breast muscle, liver, heart, intestine and abdominal fat % of broiler chicks groups. Cleary, broiler group subjected to 22 h continuous light (CL22) manifested higher dressing %, breast muscle, liver, intestine and abdominal fat % as compared by all other groups. Alternatively, non-significant differences (p<0.05) were found in the thigh, shoulder, left filet, gizzard and spleen % between all groups of broilers as affected by different lighting programs intervals (22, 20, or 20 h) whether it was continuous or intermittent (CL or IL).

### Blood biochemical parameters

Results of the lighting program’s effects on blood biochemical, blood kidney and liver function parameters are presented in Table 4. Results demonstrated non-significant effects (p< 0.05) in broiler checks blood biochemical parameters (albumin, globulin, albumin/globulin ratio, total protein, total lipids, triglycerides, and cholesterol) under different lighting programs (continuous or intermittent). Nevertheless, there were some numerical differences found among different groups whereas some blood parameters (albumin, total lipids, and cholesterol) tended to be higher in 22 h continuous light group (CL22) as compared by other groups. Also, the results clearly showed that the lighting program had no significant effect (p<0.05) on blood kidney (urea, creatinine and uric acid) and liver functions (ALT and AST) parameters of broilers as affected by different lighting programs (photoperiods whether CL or IL).

### Antioxidant and innate immunity response

Results presented in Table 5 show the oxidative stress parameters (MDA, GPx, and SOD) of broiler chicks at 42 days of age as affected by different lighting programs. Data demonstrated that different lighting programs (continuous or intermittent) significantly (p<0.01) affect oxidative stress parameters of different broiler groups. Oxidative stress parameters (MDA, GPx, and SOD) tended to be higher in broiler group, which were raised under continuous photoperiod for 22, 20, or 10 h (CL22, CL20, and CL18) than in other groups exposed to intermittent light programs (IL22, IL20, and IL18) for the same intervals. Meanwhile, decreasing photoperiod from 22 h to 20 or 18 h (whether CL or IL) significantly reduced oxidative stress parameters in broiler groups (T2, T4, and T6).

Additionally, regarding the PI and PA data presented in Table 5 showed that the lighting program (whether continuous or intermittent) significantly (p<0.01) affects the PI and PA of different broiler groups. The CL program decreased PI and PA in CL22, CL20, and CL18 groups in comparison with groups (IL22, IL20, and IL18) which exposed to the same photoperiod (22, 20, or 18 h), but in IL regimes. In addition to this, it should be noted that intermittent short photoperiod (IL18) significantly increased PI and PA values compared to other groups exposed to different lighting programs.

## DISCUSSION

### Growth performance

When the birds’ environment is taken into consideration, lighting program is one of the most critical of all environmental factors affecting performance. The lighting program affects the birds’ metabolism, which in turn is responsible for maximizing growth performance and maintaining normal physiological processes and functions. The results of the present study indicated that broiler performance in terms of BW and BWG was significantly affected by differences in lighting intervals (continuous or intermittent). Reducing lighting interval (from 22 h to 20 h or 18 h) and using intermittent light programs (IL) caused a significant reduction in BW and BWG, especially at older ages of the broiler. This reduction in BW and BWG could be explained by the reduction in time of feeding which causes a decrease in feed consumption by birds in the shortest light intervals where there was a highly positive correlation between BW and FC.

The present results are in agreement with Schwean-Lardner et al [30] who comparing different lighting schedules and demonstrated clearly that longer periods of darkness prevent regular access to feed and consequently reduce feed intake and limit growth. In the present study, the BW and BWG were significantly higher in the CL group as compared to the other lighting groups during the final stages. Also, Yang et al [31] reported that intermittent light caused a reduction in feed intake in birds and resulted in superior broiler weights in CL. However, Ingram et al [32] found that broilers reared under CL gained more weight as compared to the other exposed to intermittent or restricted light. Furthermore, Olanrewaju et al [33] reported, broiler chicks reared under the short/non-intermittent photoperiod had a significant reduction in BW and BWG from 14 to 42 of age as compared with birds reared under the long/continuous and regular/intermittent photoperiods, respectively.

In fact, broiler chickens do not feed or drink during a dark period [34]. Thus, for this reason, total FC in our study was reduced by about 5.47%, 8.06%, 11.13%, 11.39%, and 13.11% for T2, T3, T4, T5, and T6, respectively as compared with broiler group in T1 which were exposed to CL for 22 hrs. Another possible reason for feed intake reduction for birds reared under IL may be mainly due to less activity when lights were switched off which associated with secretion of melatonin from the pineal gland as reported by EI-Badry et al [35]. The results of this study in connection with feed consumption are in agreement with those of Classen et al [36] who demonstrated clearly that longer periods of darkness prevent regular access to feed and consequently reduce feed intake. Furthermore, IL has been used to restrict feed intake and improve feed efficiency which could reduce production costs as reported by Farghly and Makled [37].

Duration of lighting is a major factor affecting broiler per formance. Several investigations showed that a CL regime is not recommended as an optimal program [37,38]. The improvement in FCR of broilers exposed to shortest photoperiod and IL programs could be attributed to the reduction of physical activity, energy expenditure during dark periods, better digestion of feed, reduced nutrient requirements for maintenance and greater energy availability for growth as found by Rahimi et al [34]. While birds exposed to CL are mostly active that being associated with more stress, causing disturbance to their metabolism, and leading to a lower FCR. Additionally, another reason of the improvement in FCR could be due to lower feed consumed and lower feed waste during the dark phases. Similar results found by Mahmud et al [38] who reported that birds exposed to different IL programs consumed less feed as compared with CL. This reduction in feed consumption of birds under intermittent light treatments might have contributed to better FCR in these birds. The above result results are in full agreement with El-Slamoney et al [39] who observed that the FCR of the chick grown under various intermittent light schemes was significantly better than those grown under CL. Also, significant improvement in FCR has been observed in broilers maintained under the short intermittent program was found by Yang et al [31], who reported that FC and FCR of broilers were significantly affected by different photoperiod.

### Mortality and European Production Efficiency

Regarding the mortality rate, the application of graded levels of lighting programs on broilers indicated that extended lighting programs increased mortality, reduced livability and changed the behavior as found by Schwean-Lardner et al [30]. High mortality and low livability rate in groups raised at long continuous photoperiod may be due to the rapid growth rate of broilers which reflect in several problems, such as a high incidence of metabolic diseases (ascites and sudden death syndrome), tibial dyschondroplasia and other skeletal disorders. Moreover, the incidence of cannibalism is another major problem when light is given continuously for long periods. The intermittent light program may help to retreat this problem, as the bird will remain quiet and calm during dark hours of the light regimen. In addition to this, the metabolic disorders in broiler production could be reduced by IL programs.

Similar findings to our results were supported by Farghly and Makled [37]. Moreover, Schwean-Lardner et al [40] in an examination of the impact of graded levels of photoperiod (14, 17, 20, and 23 h) revealed the total mortality rate due to metabolic and skeletal disease decreased linearly with increasing inclusion of darkness periods. They also suggest that 7 h per day is an appropriate length of darkness for maximizing broiler welfare based on the observed health parameters. Another potential benefit of darkness is the change in bird metabolism that occurs during the dark period and the consequential restoration of tissue [41]. Also, Abbas et al [42] observed that intermittent light regimen reduced the mortality rate by three times compared to the CL regimen.

### Carcass characteristics

In regard to carcass traits, lighting programs had an effect on dressing, breast, abdominal fat, liver and intestine % in the present study, especially for the group which was exposed to 22 h (CL22). Increased dressing, breast, abdominal fat, liver and intestine % in CL22 group may be due to the increase in pre-slaughter weight which highly correlated with dressing yield. Significant correlations between pre-slaughter, carcass and breast weights were observed in this study. Similar to the current study, pre-slaughter weight was highly correlated with dressing yield in Cobb 500 and Hubbard broiler strains [43]. Reducing photoperiod (from 22 to 18 h/d) could be used successfully as a tool for decreasing abdominal fat content and improving carcass quality. This result due to less feed intake during dark periods and better efficiency in nutrient utilization.

These results were supported by the data from Rahimi et al [34] who observed a significant reduction in abdominal fat in broilers exposed to intermittent light compared with CL. Also, Oyedeji and Atteh [44] reported that there was a significant reduction in abdominal fat of broilers subjected to short photoperiod or flash program. Moreover, the obtained results are similar with the findings of Farghly and Makled [37], who reported that lighting had minimal effects on the carcass or part yields. However, they found non-significant differences in the percentages of the drumstick, femur and gizzard among all groups under IL, although the differences were significant (p<0.05) in the dressed carcass, breast, liver, and abdominal fat percentages.

### Blood biochemical indices

In general, blood biochemical parameters are used as an indicator to detect disorders due to incorrect lighting programs effecting metabolic, nutritional and welfare conditions of broilers. In the present study, there were no changes in biochemical parameters. It might be due to chronically stressed after exposure to different light programs in broiler birds. It is well known that intermittent light decreases physiological stress, improved immunity and bone metabolism [36].

The results of the present study were in full agreement with those obtained by El-Slamoney et al [39] who found that plasma total protein, total lipids, glucose, cholesterol, and triglyceride levels did not differ significantly among different lighting groups. Similarly, Farghly et al [45] found that no significant differences were observed for all blood parameters of flash lighting treated chickens and those of the control. Furthermore, Farghly et al [46] found that there was no change in plasma total protein, total lipids, cholesterol, and liver function enzymes as affected by lighting periods.

### Antioxidant status and immunity activity

Parameters of oxidative stress such as SOD, GPx, and T-AOC are indicators for assessing oxidative stress status [15]. Also, MDA is frequently used as a biomarker of oxidative stress [35]. In the present study, lower MDA, GPx, and SOD levels in broiler groups which were raised under intermittent photoperiod (IL) suggested that intermittent light regime may help to maintain the balance of oxidant-antioxidant status and may enhance the ROS scavenging by regulating the concentration of MDA, GPx and SOD. In the same way, decreasing light intervals from 22 h to 20 or 18 h. significantly reduced MDA, GPx, and SOD activity. The beneficial effect of shorter periods and intermittent light regime on MDA, GPx, and SOD may also be attributable to melatonin secretion which mainly produced during a long period of darkness. Abbas et al [42] suggest that CL is more stressful than an intermittent regime and stress generally destroys the balance of oxidant-antioxidant. These results were partially in line with previous reports indicated that CL exposure caused a significant reduction in both serum melatonin concentration and antioxidant levels in chickens [9,39] while, broiler chickens reared under IL program had better antioxidant status [46]. Additionally, melatonin decreased the homocysteine level in brain homogenates and therefore, may have a role in protecting cells from oxidative damage [47]. Also, EI-Badry et al [35] found that intermittent light decreased MDA compared to the CL group.

Phagocytic cells (non-lymphoid cells), including macro phages, neutrophils and heterophils play a crucial role in immunity which digests foreign materials, such as bacteria, viruses, denatured proteins and lipids, and apoptotic cells [27]. In this study, higher values of phagocytic cells in broiler groups which were raised under intermittent light regime, suggested that IL increased blood concentration of phagocytes. It may be due to the pineal gland that seems to control the secretion of melatonin hormone, the diurnal rhythm of non-specific immunity in chickens and then increase serum PI and PA [35]. In the same context Cardinali et al [48] reported that melatonin stimulates the production of progenitor cells for granulocytes and macrophages. It also stimulates the production of natural killer cells, CD^4+^ cells and T lymphocytes cells which enhanced by melatonin levels.

Another possible reason is that long photoperiod and CL programs decrease the opportunity for rest and sleep, thereby increasing fear reaction and physiological stress. So, birds exposed to long CL regimes have a decrease in PA and PI due to higher plasma concentrations of corticosterone, indicating the high level of physiological stress and foreign bodies which consequently cause inflammation, ultimately resulting incidence of metabolic disorders and increase MR % in the broiler. Therefore, groups reared under short intermittent light programs have lower physiological stress, improved specific and non-specific immune responses which emphasizes the importance of dark periods during the production cycle of broiler chickens [37]. The results of the present study were in line with results obtained by EI-Badry et al [35] who found that intermittent light significantly increased blood concentration of lysozyme, total white blood cells count and lymphocyte % compared to a CL group [42].

In conclusion, the results from our study revealed that, although conventional CL programs for 22 h provide an enhancement in final BW, BWG, and dressing % of broilers it still had adverse effects on FCR values, innate immunity, oxidative status, and livability ratio. In conclusion, these results suggest that reducing lighting hours up to 3L/1D seems to be more beneficial in enhancing feed efficiency, livability, antioxidant activity and immune responses as an alternative to the conventional CL programs. Finally, it is believed that a further study is needed to evaluate the effects of different photoperiods and IL programs on economic consideration in a commercial production setting.

## Figures and Tables

**Table 1:** Composition of diets fed to broilers

Items	Treatment period (0 to 42 d)	

	Starter (0 to 21 d)	Finisher (21 to 42 d)
Ingredients (%)		
Yellow corn	57.39	61.33
Soybean meal	27.00	22.80
Palm oil	2.20	2.80
Corn gluten meal	8.80	6.00
Wheat bran	--	3.00
Dicalcium phosphate	2.30	2.09
Ground limestone	0.70	0.62
Choline chloride	0.05	0.05
DL-methionine	0.105	0.075
L-lysine	0.39	0.36
Salt	0.40	0.20
Threonine	0.17	0.17
Vitamin-mineral premix^1)^	0.50	0.50
Calculated chemical analysis		
ME (kcal/kg)	2,900	3,200
Crude protein (%)	23.0	21.5
Non-phytate P (%)	0.48	0.44
Calcium (%)	0.96	0.88
Digestible lysine (%)	1.28	1.15
Digestible methionine (%)	0.60	0.54
Digestible sulfur amino acids (%)	0.95	0.86
Digestible threonine (%)	0.86	0.77

1) Vitamin-mineral premix contains per kg: vitamin A, 2,400,000 IU; vitamin D, 1,000,000 IU; vitamin E, 16,000 IU; vitamin K, 800 mg; vitamin B_1_, 600 mg; vitamin B_2_, 1,600 mg; vitamin B_6_, 1,000 mg; vitamin B_12_, 6 mg; niacin, 8,000 mg; folic acid, 400 mg; pantothenic acid, 3,000 mg; biotin 40 mg; antioxidant, 3,000 mg; cobalt, 80 mg; copper, 2,000 mg; iodine, 400; iron, 1,200 mg; manganese, 18,000 mg; selenium, 60 mg; zinc, 14,000 mg.

**Table 2:** Effect of lighting program on growth performance, livability and European Production Efficiency in broiler

Parameters	Treatments						SEM	p-level

	CL22	IL22	CL20	IL20	CL18	IL18		
IBW (g)	40.2	40.5	40.6	40.1	40.3	40.2	0.34	NS
FBW (g)	2,723^a^	2,575^b^	2,538^c^	2,492^d^	2,474^d^	2,416^e^	3.92	^***^
FC (g)	4,241^a^	4,009^b^	3,899^c^	3,769^d^	3,758^d^	3,685^e^	2.20	^***^
BWG (g)	2,683^a^	2,534^b^	2,497^c^	2,452^d^	2,434^d^	2,376^e^	3.05	^**^
FCR	1.58^a^	1.58^a^	1.56^ab^	1.54^b^	1.55^b^	1.54^b^	0.02	^*^
Mortality (%)	3.41^a^	2.61^ab^	3.02^a^	2.01^b^	1.10^c^	0.40^c^	0.21	^**^
Livability (%)	96.6^c^	97.4^bc^	97.0^bc^	98.0^b^	98.9^a^	99.6^a^	0.23	^**^
EPEF	396.01^a^	378.13^b^	375.97^c^	377.1^bc^	375.1^c^	372.6^d^	4.59	^*^

CL, continuous lighting program; IL, intermitted lighting program; SEM, standard error of the mean; IBW, initial body weight; NS, not significant; FBW, final body weight; FC, feed consumption; BWG, body weight gain; FCR, feed conversion ratio; EPEF, European Production Efficiency.

a–e Means within row followed by different superscripts are significantly different.

* p<0.05,

** p<0.01,

*** p<0.001.

**Table 3:** Effect of lighting program on carcass traits and internal organ of broilers

Parameters	Treatments						SEM	p-level

	CL22	IL22	CL20	IL20	CL18	IL18		
Carcass traits (%)								
Dressing	72.84^a^	70.41^b^	70.49^b^	70.44^b^	69.94^b^	70.92^b^	0.54	^*^
Abdominal fat	2.05^a^	1.87^ab^	1.70^b^	1.66^b^	1.65^b^	1.58^b^	0.12	^*^
Breast	28.08^a^	26.48^ab^	26.22^ab^	25.94^b^	25.76^b^	26.77^ab^	0.52	^**^
Thigh	16.47	16.28	15.75	16.53	15.71	16.26	0.48	NS
Shoulder	4.57	4.19	4.07	4.05	4.29	4.58	0.19	NS
Left filet	11.53	11.13	10.55	10.74	10.52	11.34	0.34	NS
Internal organ (%)								
Liver	4.38^a^	3.96^b^	3.95^b^	3.84^b^	3.82^b^	3.89^b^	0.86	^*^
Gizzard	3.09	2.88	2.76	2.83	2.89	3.02	0.08	NS
Heart	0.91^a^	0.83^b^	0.86^ab^	0.90^ab^	0.90^ab^	0.92^a^	0.02	^**^
Spleen	0.21	0.20	0.20	0.21	0.20	0.20	0.01	NS
Intestine	4.08^a^	3.66^b^	3.65^b^	3.54^b^	3.52^b^	3.59^b^	0.18	^*^

CL, continuous lighting program; IL, intermitted lighting program; SEM, standard error of the mean; NS, not significant.

a,b Means within row followed by different superscripts are significantly different;

* p<0.05,

** p<0.01.

**Table 4:** Effect of lighting program on blood biochemical parameters in broiler

Item	Treatments						SEM	p-level

	CL22	IL22	CL20	IL20	CL18	IL18		
Parameters								
Total protein (g/dL)	3.48	3.44	3.48	3.52	3.36	3.40	0.07	NS
Albumin (g/dL)	1.85	1.79	1.83	1.78	1.80	1.81	0.04	NS
Globulin (g/dL)	1.63	1.66	1.64	1.74	1.56	1.58	0.06	NS
A/G ratio	1.15	1.09	1.13	1.03	1.17	1.16	0.05	NS
Total lipid (mg/dL)	538.2	511.1	521.2	533.3	453.4	484.6	1.17	NS
Triglyceride (mg/dL)	137.5	136.9	130.9	133.6	128.1	127.8	0.53	NS
Cholesterol (mg/dL)	150.2	143.4	149.8	153.7	135.3	143.3	1.56	NS
Liver function								
AST (U/L)	97.80	96.10	93.90	93.70	92.40	91.40	2.48	NS
ALT (U/L)	21.20	19.50	20.01	19.41	20.21	17.80	0.51	NS
Kidney function								
Creatinine (mg/dL)	0.44	0.45	0.46	0.45	0.45	0.46	0.01	NS
Uric acid (μmol/L)	414.5	411.2	411.3	414.2	414.3	410.5	2.41	NS
Urea (mmol/L)	5.40	5.43	5.46	5.47	5.51	5.45	0.07	NS

CL, continuous lighting program; IL, intermitted lighting program; SEM, standard error of the mean; NS, not-significant; A/G ratio, albumin/globulin ratio; AST, aspartate aminotransferase; ALT, alanine transaminase.

**Table 5:** Effect of lighting program on antioxidant and immunity response of broilers

Parameters	Treatments						SEM	p-level

	CL22	IL22	CL20	IL20	CL18	IL18		
Antioxidant activity								
MDA (μmol/L)	2.15^a^	1.72^b^	1.85^ab^	1.68^b^	1.82^ab^	1.57^b^	0.11	^**^
GPx (U/g Hb)	22.10^a^	18.80^bc^	20.01^a^	17.20^c^	19.10^a^	15.40^d^	0.59	^**^
SOD (U/g Hb)	81.40^a^	72.50^ab^	75.90^a^	60.30^bc^	74.00^ab^	57.01^c^	0.89	^***^
Innate immunity status								
Phagocytic index	1.57^ab^	1.58^ab^	1.58^ab^	1.69^a^	1.50^b^	1.72^a^	0.05	^**^
Phagocytic activity	15.90^b^	15.97^b^	16.01^b^	16.40^ab^	15.55^b^	16.95^a^	0.29	^**^

CL, continuous lighting program; IL, intermitted lighting program; SEM, standard error of the mean; MDA, malonaldehyde; GPx, glutathione peroxidase; SOD, super oxide-dismutase.

a–d Means within row followed by different superscripts are significantly different;

** p<0.01;

*** p<0.001.
